# Evolution of laser technology for automotive LiDAR, an industrial viewpoint

**DOI:** 10.1038/s41467-024-51975-6

**Published:** 2024-09-03

**Authors:** Dong Liang, Cheng Zhang, Pengfei Zhang, Song Liu, Huijie Li, Shouzhu Niu, Ryan Z. Rao, Li Zhao, Xiaochi Chen, Hanxuan Li, Yijie Huo

**Affiliations:** Vertilite Co. Ltd, Wujin District, Changzhou, China

**Keywords:** Lasers, LEDs and light sources, Semiconductor lasers, Diode lasers

## Abstract

Liang et al. present an industrial perspective on the evolving landscape of laser technology used in advanced LiDAR systems. The authors discuss recent trends, practical considerations within the industry, current challenges, and potential solutions, explicitly focusing on VCSEL/AR-VCSEL-based technologies and their strong potential for commercial LiDAR applications.

## Teaser

From an industry perspective, the past decade has been a whirlwind of innovation in automotive light detection and ranging (LiDAR). Numerous laser technologies and system solutions have been fiercely competing for market share. However, recent trends suggest a growing convergence on vertical-cavity surface-emitting laser (VCSEL) and antireflective VCSEL (AR-VCSEL) based solutions. This commentary, rooted in the practical realities of the industry, examines the historical trajectory of industrial laser technology for commercial automotive LiDAR. It specifically focuses on the recent applications of VCSEL/AR-VCSEL technologies and their future prospects.

## An introduction to LiDAR

LiDAR (Light Detection and Ranging) was invented in the 1960s by Hughes shortly after Theodore Maiman and his team demonstrated the first ruby laser^1,2^. It was initially applied in meteorology, ocean sensing, and topographic mapping. In 1971, NASA integrated a LiDAR, known as lunar Laser Ranging RetroReflector (LRRR) in Apollo 15 to map the moon’s surface, and later extended its use in the spacecraft bound for Mars and Mercury^3,4^. It was not until the 2010s that LiDAR started to be applied in commercial automobiles. In the 2020s, the automotive LiDAR has become popular in high-end electric cars. Offering real-time point-cloud images enriched with object depth and velocity data, LiDAR serves as a pivotal component for both assisted driving and self-driving. There are over a hundred LiDAR companies globally. North America pioneered in commercializing automotive LiDAR with Velodyne supplying mechanical spinning LiDAR HDL-64E to numerous self-driving companies in Silicon Valley during the first half of the 2010s^5^. This region also hosts the most LiDAR company IPOs. Asia, particularly amidst the surge in smart electric vehicle development, has witnessed a notable emergence of LiDAR companies in recent years. In contrast, Europe is marked by the dominance of traditional giants. The industry experienced some turbulence in 2022, marked by the bankruptcy filings of LiDAR pioneers Quanergy and Ibeo. However, in 2023, there was a surge in LiDAR sales driven by intense competition between Chinese EV makers. Today, key players in the global LiDAR market include Valeo in Europe, Luminar and Ouster in North America, and Hesai, RoboSense, Seyond, and Innoviz in Asia.

A typical automotive LiDAR system comprises a scanning laser, a receiver, associated optics, and integrated driver and processor circuits. It collaborates with cameras, sensors, and the position and navigation system. Functionally, automotive LiDAR falls into two main categories: primary LiDAR, responsible for long-range forward perception, and supplementary LiDAR, used for peripheral environment sensing. Together, they can achieve 360° omnidirectional perception, eliminating blind spots. The required detection range for primary LiDAR varies globally from 150 m to 350 m, influenced by several factors: diverse vehicle speed limits, the targeted level of driving automation (as classified by the Society of Automobile Engineers as six levels)^6^, and regional regulations^7^.

Based on the detection method, LiDAR technology can be classified into two types, namely frequency modulated continuous wave (FMCW) and time of flight (ToF). FMCW utilizes the mixing of returned light with frequency-modulated transmitted light to ascertain the distance and velocity of a moving object^8^. ToF, on the other hand, determines the distance by calculating the time interval between the emitted pulse and the returned pulse. ToF is also the earliest technology used for LiDAR, e.g. in LRRR. Currently, most LiDAR manufacturers are favouring ToF technology due to its simplicity and lower cost. As a result, the following discussion will primarily focus on ToF and related laser technology.

## Laser technologies for commercial LiDARs across different ranges

There are numerous exciting innovations of laser technologies integrated with advanced optics^9–15^. These innovations, especially the nanophotonics solutions, offer a higher degree of integration of laser and scanning, enable further miniaturisation and hold promise for the long-term future of LiDAR systems. This commentary will focus on laser solutions successfully utilised in commercial automotive LiDARs. We will analyse the evolving dynamics and trends shaping this landscape over the next few years.

Table 1 shows the various laser technologies currently employed in commercial LiDAR systems for different ranges. The 1550 nm fibre laser stands out for its efficacy in long-distance detection, attributed to the higher power limit for eye safety at this wavelength. Typical LiDAR products employing 1550 nm ToF solution include Luminar Iris and Seyond Falcon. While this solution excels in detection range and resolution, it faces significant challenges related to high cost for both lasers and the InGaAs detectors, heat dissipation issues due to high power, reliability risk and large physical dimensions.

**Table 1:** Mainstream laser technologies for commercial LiDARs across different ranges

Laser type	Suitable range				Cost	Size
	Longer distance	Long distance	Mid distance	Short distance		
	200–400 m	100–200 m	30–100 m	0–30 m		
Fibre laser 1550 nm					High	Bulky
EEL 905 nm					Medium	Medium
VCSELs 905/940 nm multijunction					Low	Compact
AR-VCSELs 905/940 nm multijunction					Low	Very compact

In the realm of mid to long-range LiDAR, the 905 nm EEL (edge emitting laser) offers a more economical solution in both cost and size compared to fibre lasers. OSRAM’s 905 nm triple-junction EELs, known for their temperature stability, combined with MEMS mirrors have achieved success in first-generation hybrid LiDAR systems. The range of 905 nm LiDAR has been significantly improved in recent years thanks to the higher efficiency detectors^16,17^. Sony’s IMX459 stacked SPAD depth sensor with a photon detection efficiency of 24% released in late 2021 has become one of the most popular sensors for LiDAR^18–20^.

VCSELs were first applied in short-distance LiDAR and 3D sensing for phones and consumer devices, pioneered by Philips, Lumentum, Coherent (II–V and Finisar), and AMS (Princeton and Vixar)^21,22^. VCSELs have many advantages compared to EELs: 1. Flexible illumination, such as 1D/2D addressable arrays. 2. Intrinsic temperature stability (0.07 nm/°C). 3. Circular beams for simple optics. 4. Easier packaging. 5. Extra redundancy in the reliability of an array instead of a single emitter. 6. Cost-effectiveness, with 6-inch GaAs foundries well established in smartphone 3D sensing mass production. The only disadvantages were their power density and brightness. The advent of multijunction technology has significantly enhanced their power density and power conversion efficiency (PCE), overcoming a previous bottleneck in their application for mid to long-range LiDAR, e.g. Lumentum’s 905 nm 5-junction VCSELs for Hesai AT128^23^.

A recent emerging competitive technology in the LiDAR landscape is Antireflective VCSELs (AR-VCSELs)^24^, which marks a notable breakthrough in decreasing divergence and enhancing brightness. The introduction of AR-VCSELs extends the detection range and resolution of 905 nm and 940 nm based LiDAR even further, covering all the necessary ranges for automotive LiDAR. Although only recently published, AR-VCSELs were developed in 2021 and have already been adopted in commercial long-range LiDARs.

## Coevolution of scanning methods and laser technologies

**Fig. 1:**
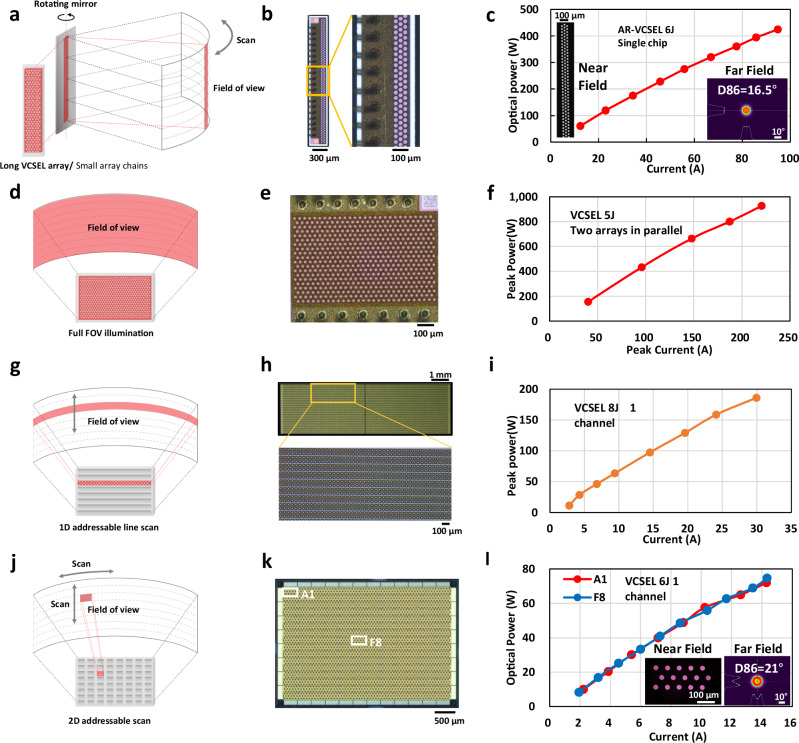
Four types of VCSEL-based LiDAR scanning schemes and their performance. **a** Hybrid scanning with a long and narrow array or a chain of smaller arrays replacing the need for vertical scanning, and a rotating mirror handling horizontal scanning. **b** Image of a single 6-junction AR-VCSEL narrow array for hybrid scanning. **c** Light output power vs the driving current (LI curve) of the long AR-VCSEL array, as well as its near-field (NF) and far-field (FF) images, tested at 50 kHz repetition rate and 3ns pulse width at the ambient temperature of 50 °C. **d** Flash illumination where the whole FOV is illuminated at once. **e** Image of a 5-junction VCSEL array used for flash illumination. **f** LI curves of two VCSEL arrays in parallel to provide high power for flash illumination, tested at 100 kHz repetition rate and 3 ns pulse width at room temperature. **g** 1D addressable line scan with a large chip integrating a group of narrow arrays that can be independently turned on and off. **h** Image of a 1D addressable 8-junction VCSEL array. **i** LI curve of a single channel from the 1D addressable array tested at 20 kHz and 4 ns pulse width at room temperature. **j** 2D addressable matrix scan by electronically activating specific columns and rows to locate the section that needed to be illuminated. **k** Image of a 2D addressable 6-junction VCSEL array. The upper-left corner zone is labelled as Zone A1, and the zone near the middle of the array is labelled as Zone F8. **l** LI curves of the 2D addressable VCSEL array Zone A1 and F18, as well as NF and FF images of Zone F18, tested at 20 kHz repetition rate and 5 ns pulse width at the ambient temperature of 50 °C.

There are three types of commercial automotive LiDARs based on the scanning method: Mechanical LiDAR (involving the movement of lasers, lenses, and sensors), Hybrid Solid State LiDAR (in which only the scanning MEMS/mirror moves), and All-Solid-State LiDAR (with no mechanical movements, as the scanning beam is controlled electrically). Fig. 1 shows four types of VCSEL-based LiDAR scanning schemes, including one type of hybrid scanning (Fig. 1a), and three types of solid-state scanning (Fig. 1d, g, and j). There are pros and cons for each of the solutions^23^. Other scanning methods include optical phase arrays (OPA)^8,10,13,14^, focal plane switch array^25^, acousto-optic beam steering^26^, planar-lens^11^, MEMS-integrated metasurfaces^10^, beam steering metasurfaces^9,12^, liquid crystal metasurface (LCM) devices^10^, etc. Among them, OPA and LCM are commercially available yet to achieve mass production, while others are still in the research phase.

## Hybrid solid state: EEL vs AR-VCSELs

Pure mechanical LiDAR has nearly phased out of existence in Level 2 (L2, partial driving automation) and Level 3 (L3, conditional driving automation) advanced driver assistance systems (ADAS) as the hybrid solid-state solution takes centre stage in the industry.

Hybrid solid-state LiDAR manufacturers initially combined a point source (such as a 1550 nm fibre laser or 905 nm EEL) with 2D MEMS or mirrors. A recent popular solution features a solid-state source electrically scanning in one direction and a 1D polygon/mirror scanning in the other. This solid-state source consists of either a series of small VCSEL/AR-VCSEL chips (e.g. Hesai AT128) or a chain of VCSEL/AR-VCSEL narrow arrays. This evolution eliminates the need for precise alignment between lasers and MEMS, addresses field of view (FOV) issues associated with MEMS (e.g. Robosense M1 requiring five EEL modules to achieve a 120° FOV), and reduces the number of motors from two to one.

Figure 1b shows a 2.6 mm long and 85 μm narrow (emission area) array of 6-junction AR-VCSEL, with a peak output power of 400 W at a 16-degree divergence in D86 (defined as the angle of the D86 beam, whose width is the diameter of the circle that is centred at the centroid of the beam far field profile and contains 86% of the beam power), as shown in Fig. 1c. The low divergence multi-junction AR-VCSEL narrow array maintains a small beam parameter product (*BPP*)^27^ along its short side, enabling high horizontal resolution while enhancing total power by extending its vertical length (vertical resolution, in this case, will be determined by the pixel size and density of the receiver).

While Fig. 1 primarily focuses on VCSEL-based solutions, EELs and fibre lasers can also be integrated into hybrid solid-state LiDAR systems. Notably, EELs, combined with 2D-MEMS technology, have achieved commercial success, as evidenced by LiDAR products like Robosense M1 and MX. In the coming years, we anticipate a competition between EEL and AR-VCSEL-based solutions. Low-cost EEL-based LiDAR reduces the number of EELs to a minimum and addresses the angle coverage problem with additional lenses. AR-VCSEL, on the other hand, will have more upside in power density and brightness while shrinking the device area. Performance-wise, EEL-based LiDARs typically have a range limit of 200 m, whereas AR-VSCEL-based LiDARs with the current 6-junction technology already surpass this range, and advancements to 8-10 junctions promise to push it further towards 300–400 m. Additionally, the generally lower cost of VCSELs compared to EELs suggests that AR-VCSELs may hold significant long-term advantages.

## The “holy grail”: all-solid-state LiDAR

All-solid-state LiDAR eliminates moving parts and replaces mechanical scanning with electrical scanning. Among the commercially viable solutions are: VCSEL with defocus lenses for flash illumination (Fig. 1d), and 1D/2D addressable VCSEL with defocus lenses (Fig. 1g, j). Additional options include VCSEL/EEL with LCM by Lumotive, and FMCW EEL with OPA LiDAR, demonstrated by Quanergy, Aeva, LightIC, Scantinel Photonics, etc. These solutions are yet to achieve scaled production, while addressable VCSEL arrays for LiDAR are gradually entering the mass production phase.

Flash VCSELs initially found applications in ToF cameras on smartphones, offering flood illumination of the entire field of view. However, their detection range is limited, usually for short distances. Medium-to-long distance LiDARs utilise cyclic scanning with 1D and 2D addressable VCSEL arrays. As shown in Fig. 1h, i, 1D technology can be considered a cluster of VCSEL narrow arrays with individual anodes with a common cathode. 2D addressable VCSEL array matrix (Fig. 1k) allows individual control of both anodes and cathodes, providing even more flexibility in the illumination strategies. However, their metal interconnect adds complexity to the fabrication and faces slightly more challenges compared to the 1D solution. Figure 1l shows the LI performance, NF and FF images of individual zones from a standard 2D addressable VCSEL array.

Most all-solid-state LiDAR solutions being developed today are targeting short to mid-range first. We believe that once proven in shorter distances, all-solid-state longer-distance LiDAR will emerge, where AR-VCSEL will likely play a crucial role. Rapidly progressing in both technology readiness and cost-effectiveness, the VCSEL solution for all-solid-state LiDAR is establishing itself as the most competitive candidate to achieve the “holy grail”.

## Key requirements for future laser technologies in LiDAR

The following section explores key requirements and future directions on lasers for LiDAR, including high power density, high PCE, good beam quality, robust reliability, and cost-effectiveness^16^.

## Power density and PCE

**Fig. 2:**
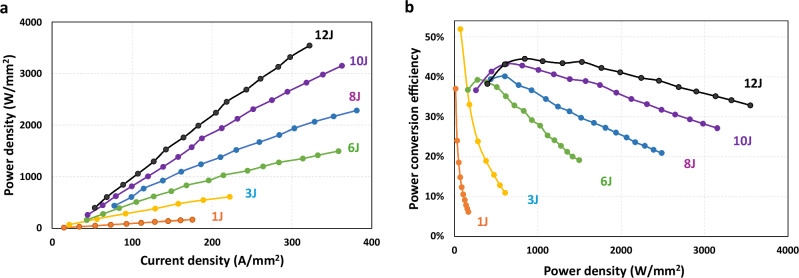
VCSEL power and efficiency scale with the number of junctions. **a** The power density vs the current density curves for VCSEL arrays with junction numbers ranging from 1 to 12; **b** the PCE for 1–12 junction VCSELs. All devices are tested at a 20 kHz repetition rate and 3 ns pulse width at the ambient temperature of 65 °C. 1 J and 3 J samples are 575 μm × 479 μm rectangular array, while 6 J, 8 J,10 J, and 12 J are 250 μm diameter circular array.

Higher peak power enables greater signal-to-noise ratios and longer detection ranges for LiDAR. A greater number of junctions ensures a higher external quantum efficiency, directly proportional to the junction count. Consequently, this results in a higher power density at the same driving current (Fig. 2a), and a greater PCE for the same optical power (Fig. 2b). For current VCSEL-based LiDARs in the market, the number of junctions is 5–6 this year, and there is a likelihood of an increase by 2 every 18 months, like Moore’s Law. For research and development purposes, we have experimentally demonstrated small-divergence AR-VCSELs up to 14 junctions^24^. Theoretically, there is no upper limit for the number of junctions. However, in practical terms, incorporating more junctions may present challenges in terms of thick epitaxial growth, high aspect ratio trench/mesa etching and coating in fabrication, and reliability concerns at higher power density and material stress.

## Beam parameter product (*BPP*)

*BPP* is defined as the product of a laser beam’s divergence angle *θ* (half-angle) and the radius of the beam at its narrowest point *r* (the beam waist). Mathematically, it is expressed as

1 $${BPP}=\frac{\theta }{2}\times r=\frac{\lambda }{\pi }\, {M}^{2}$$

Where *M* ^2^ denotes the beam quality and λ is the wavelength. For the ideal Gaussian beam with *M* ^2^ = 1, *BPP* is at its minimum of λ/π. The product of *BPPs* along x and y is inversely proportional to the brightness of a laser when *θ* is small.

For a LiDAR system with sufficient sensor resolution, the spatial resolution limit is approximately equal to the size of the laser beam on the illuminated object after collimation, which can be expressed as

2 $$\Delta x=4\times \frac{{BPP}}{D}\times R$$

Where *D* is the diameter of the transmitting lens, and *R* is the distance to the target. Therefore, the smaller the *BPP*, the better the resolution for the same optics. Smaller *BPP* or *M* ^2^ allows the use of smaller lenses, facilitates longer ranges, and enhances resolution.

**Fig. 3:**
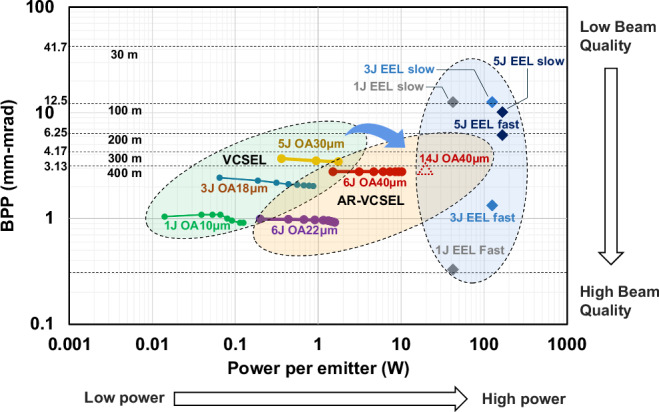
The beam parameter product (*BPP*) vs laser power. Round dots are for single emitter VCSELs (1 J, 3 J, and 5 J) and AR-VCSELs (6 J) at various oxidation aperture diameters and driving currents. The 14 J OA 40 μm is the predicted value. Diamond dots are *BPP* along the fast and slow axis in EELs (1 J, 3 J, and 5 J).

As shown in Fig. 3, the *BPP* for an EEL differs between its fast and slow axes^16,27^. The fast axis has a 3×–8× larger angle but is typically 10×–1000× smaller in diameter compared to the slow axis, resulting in a smaller *BPP* for the fast axis. Although multijunction EEL provides higher single-emitter power, as the number of junctions increases from 1 to 5, the *BPP* of the fast axis is significantly traded off, thereby limiting resolution at long distances.

VCSEL/AR-VCSEL’s circular aperture ensures a symmetrical *BPP* at the single emitter level. Their oxidation aperture (OA) size determines the radius of the beam. A larger OA allows higher power output while maintaining the driving current density but conversely increases both divergence and *BPP*. Therefore, the OA size must be picked carefully to balance power and *BPP* requirements. Although multijunction helps deliver sufficient power, traditional VCSELs struggle with *BPP* once the number of junctions reaches 5 or above and OA reaches over 20 μm. AR-VCSELs with exceptional *BPP* and *M* ^2^ enable longer distances and higher resolutions than traditional VCSELs. As shown in Fig. 3, a 6 J AR-VCSEL emitter with 40 μm OA has a lower *BPP* than a traditional 5 J VCSEL with 30 μm OA, but five times the power output. A 6 J AR-VCSEL emitter with 22 μm OA shows the same level of power but a quarter of the *BPP* compared to the 5 J VCSEL.

To echo Table 1’s range, we mark the *BPP* requirement to achieve the spatial resolution of 10 cm at 30 m, 100 m, 200 m, 300 m, and 400 m, assuming a collimation lens diameter of 5 cm. For example, 10 cm spatial resolution at 200 m is about 0.03° in angular resolution requiring a *BPP* of 6.25, which allows up to two columns of 40 μm AR-VCSEL emitters or up to six columns of 20 μm AR-VCSEL emitters to provide adequate power at the same time. The trend of progression from traditional VCSEL to AR-VCSELs, moving to the right and down, aligns with the anticipated trajectory for future long-range LiDAR lasers.

## Reliability

**Fig. 4:**
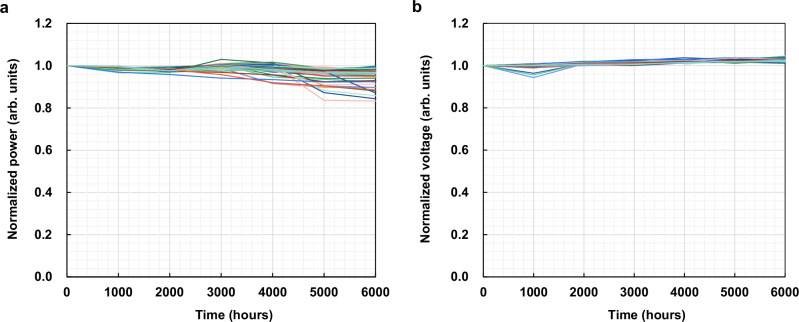
HTOL reliability test of 250 um-diameter 6 J AR-VCSEL circular arrays under accelerated conditions. **a** Normalized power over time. **b** The normalized voltage over time. (HTOL condition: 5 ns pulse width, 600 kHz repetition rate at 85 °C environmental temperature and ~130 °C junction temperature with initial peak power ~50 W. A normalized power degradation of over 20% is considered a failure).

Safety first. To ensure the reliable operation of LiDAR throughout a vehicle’s lifetime, the lasers must pass the automobile standard reliability test, AEC-Q102, which includes a high temperature operating lifetime (HTOL) of 1000 h, HTOL under 85 °C and 85% humidity environment of 1000 h, low-temperature operating lifetime (LTOL) of 500 h, powered/unpowered temperature cycling, harmful gas test, Dew test, ESD test, etc. While AEC-Q102 is the basic reliability standard for VCSELs used in automotive LiDAR, every LiDAR manufacturer sets its own standard which is normally higher than the AEC-Q102 standard, including rigorous requirements for the Failures in Time (FIT) rate, which is the number of failures expected in one billion device-hours of operation. Figure 4 shows that 42 units of AR-VCSEL array chips all survived the 6000 h HTOL test, well above the AEC-Q102 requirement. This is equivalent to over 300 years at the customer’s field use condition, sufficient in redundancy. In addition to the long-term ageing study, tens of thousands of AR-VCSEL array chips have been subjected to the FIT study. Our test shows 0 failure for nearly 3 billion equivalent device hours, and FIT is <0.8 at a 90% confidence level. Although the higher power density requirement in future LiDAR may add stress to the lifetime of AR-VCSEL, it seems to have sufficient redundancy to embrace the challenges. A similar lifetime has been achieved on regular multijunction VCSEL arrays as well.

## Cost down

In the short term, we anticipate the rapid replacement of 1550 nm fibre lasers with 905 nm/940 nm VCSELs within the next few years. Nine hundred five nanometre EELs with MEMS solution will likely hold the ground for a while competing in cost with VCSEL solutions. A lower-cost alternative solution for 1550 nm lasers is InP-based high-power EELs, which, however, still appear more expensive than GaAs-based lasers.

In the ongoing development of LiDAR technology, the primary focus is on balancing distance and cost. Once the 200–300 m distance requirement is met, the added benefits of higher resolution and an even larger range, while desirable, are somewhat limited compared to the urgency for reduced costs. Over the past decade, LiDAR costs have plummeted from over $10,000 to the current range of $500 to $1,000. This downward trend is expected to continue, potentially reaching the $100 range in the coming years. Global LiDAR penetration now is in the order of 0.5% of all passenger cars sold. We anticipate a surge in this figure to exceed 10% as the selling price of LiDAR approaches $100.

VCSELs, recognized for their small area/power ratio and established cost-effectiveness and reliability in billion-unit consumer markets, are well-positioned to compete favourably in cost reduction against fibre lasers and EELs. We anticipate that VCSEL and AR-VCSEL-based LiDAR could potentially achieve cost parity with 4D mm-wave radar in the future while offering significantly enhanced angular resolution and accuracy.

## Summary

It is evident that LiDAR, alongside visual cameras, is becoming increasingly necessary for ADAS L3 and above due to its superior accuracy in object localisation and minimal influence by ambient light. As advancements in ADAS L3 consumer vehicles accelerate, automakers are increasingly embracing LiDAR technology. Laser manufacturers are making significant strides in developing cost-effective and reliable solutions to achieve high power density, high energy conversion efficiency, and high beam quality. While long-range LiDARs initially use 1550 nm high-power fibre lasers and 905 nm EEL solutions, these solutions gradually face challenges in manufacturing costs. VCSEL technology, known for its cost-effectiveness in the smartphone and consumer electronics market, is continuing its evolution and finding new applications in automotive LiDAR, in both the hybrid-solid-state and all-solid-state solutions. Multijunction and AR-VCSEL technologies address the historical limitations of VCSELs by offering increased power density, superior beam quality and higher brightness. These advancements significantly extend the range of VCSEL-based LiDARs and position VCSEL technology as a strong contender in the cost-competitive automotive LiDAR market.

## Data Availability

The data that support the findings of this study are provided in the main text. All the relevant data are available from the corresponding author upon request.
